# Effect and Interaction of β-Lactoglobulin, Kappa Casein, and Prolactin Genes on Milk Production and Composition of Awassi Sheep

**DOI:** 10.3390/ani9060382

**Published:** 2019-06-21

**Authors:** Khaleel Jawasreh, Ahmad Al Amareen, Pauline Aad

**Affiliations:** 1 Department of Animal Production, Jordan University of Science and Technology (JUST), Irbid 22110, Jordan; 2 Livestock Directorate, National Agriculture Research center (NARC), Albaqa’a 19381, Jordan; ahmad_alathamna@yahoo.com; 3 Department of Sciences, Notre Dame University Louaize, Zouk Mosbeh 1211, Lebanon; paad@ndu.edu.lb

**Keywords:** Awassi sheep, gene interaction, milk components

## Abstract

**Simple Summary:**

Beta-lactoglobulin (*β-LG*), prolactin (*PRL*), and Kappa casein (*CSN3*) all contribute to the determination of milk production and composition, but have not been assessed in local Awassi sheep. Therefore, our aim was to analyze the contribution of these genes in milk production and composition traits in commercial Awassi ewe population by genotyping and sequencing these genes. Our results showed the prevalence of the different variations (alleles) of the tested genes in the Awassi population, and no association among *β-LG* and *CSN3* polymorphic genotypes and milk production, or *PRL* and fat%. Also, all 3 genes help determine the milk production potential of Awassi ewes and help assess milk components, and thus can be used in breeding programs to select for milk potential.

**Abstract:**

A participatory animal-breeding program was applied to 9 commercial Awassi sheep flocks in Jordan. This study aimed to assess the influence of Beta-lactoglobulin (*β-LG*), Prolactin (*PRL*), and Kappa casein (*CSN3*) genes, genotypes and their interaction on milk production and composition traits of 167 genotyped Awassi ewes via Polymerase Chain Reaction (PCR) followed by sequencing. Allele frequencies for the two variants were 0.42 and 0.58 for *β-LG*, 0.82 and 0.18 for *PRL*, and 0.92 and 0.08 for *CSN3*. No association was found among *β-LG* and CSN3 polymorphic genotypes with milk production traits. However, ewes with *PRL* AA genotype showed higher milk production, *β-LG* AB was associated with lowest fat%, high solid not fat (SNF)%, protein%, and lactose%. *β-LG* BB was associated with highest milk density. *PRL*, *β-LG*, and *CSN3* polymorphic genotypes were differentially associated with milk production and component traits. Furthermore, *β-LG* × *PRL* interaction showed the highest milk production and fat%; *β-LG* × *PRL* recorded the highest SNF%, protein%, lactose%, and milk density, while the *PRL* × *CSN3* had the highest fat% and SNF%. The enhancing effects of these gene interactions can be incorporated in Awassi breeding programs to improve milk production and composition.

## 1. Introduction 

The dairy industry is a crucial cog in the agricultural economy of many countries. Therefore, milk yield and its components are among the major goals targeted by animal geneticists [1]. Recently, increasing interest in sheep milk has led to extensive studies aiming to explore the local sheep breed’s genetic potential in milk production [2]. Awassi is the most common breed of sheep in Middle East countries where its products, including meat, milk, and wool, play an important socioeconomic role in Jordan. Awassi lambs are profitable and the milk of Awassi ewes is a commodity valued by farmers for its ability to cover annual production costs. For this reason, Awassi daily milk production has been enhanced via genetic selection from 0.5 kg to 1.2 kg [3]. Meanwhile, milk production and composition in Mediterranean countries was observed to fluctuate within and between flocks and countries as a result of different environmental factors and variations within and between Awassi genotypes [4,5,6,7,8,9]. In the past, animal breeders have made effective efforts to improve the performance of livestock by artificial selection and breeding to increase the frequency of certain desirable traits. This only involved the direct use of phenotypic observations without any knowledge of the underlying molecular information. Therefore, there is a need to adopt methods of selection based on genomic queues [10].

Milk protein polymorphisms are of great importance in the dairy industry and animal-breeding programs due to its association with quantitative and qualitative milk traits and its potential use in genetic selection programs of dairy sheep breeds [11]. Many studies with diverse breeds of dairy sheep have indicated Beta-lactoglobulin (*β-LG*), Prolactin (*PRL*) and Kappa casein (*CSN3*) as promising candidate genes for milk production and composition [2,12,13,14,15,16]. 

Beta-lactoglobulin, coded by the *β-LG* gene, is synthesized by the secreting cells of the mammary gland. *β-LG* is the primary whey protein in ruminant’s milk and accounts for approximately 17 to 22% of total milk proteins [15]. The *β-LG* gene is located on ovine chromosome 3 [17], and exon number 2 of *β-LG* revealed three allelic polymorphisms (A, B and C) based on different amino acid changes. The alleles A and B (Tyr/His) differ at the amino acid position 20 [18] and the genetic variant C differs from variant A by an amino acid exchange at position 148 (Arg/Gln) (GenBank accession No. X12817). The most common genetic variants detected in all studied sheep breeds are A and B, while the variant C is regarded as rare, and only found with low frequencies in Carranzana, Black Merino and White Merino breeds [15]. Subsequent studies have demonstrated the *β-LG* gene’s polymorphic effect on milk components including yield, protein, fat, and lactose content [14,19,20] as well as the impact of protein genetic variants on the properties of milk [2].

Prolactin, coded by the *PRL* gene, is a lactogenic hormone found in many species. The *PRL* gene plays a key role in the development of the mammary gland and milk secretion; its depletion in sheep provokes a severe reduction in milk production [21], suggesting that *PRL* is a functional candidate gene contributing to variations in milk production. Many researchers suggested the association of *PRL* gene polymorphisms with milk yield and composition traits in different dairy sheep breeds [13,14,16,22]. The *PRL* gene is in a region of the ovine chromosome 20 with putative quantitative trait locus (QTL) for milk yield and composition [23]. Thus, PRL is primarily responsible for the synthesis of fat, proteins, and all other major components of milk [24]. These characteristics make *PRL* a strong candidate gene for milk traits, and potentially be used as a positional marker gene associated with milk yield and composition. 

The four caseins (*αS1-, αS2-, β- and κ-casein*) are the major proteins in sheep milk, accounting for about 80% of total protein in milk [24]. Among the 4 caseins, *κ-casein* (*CSN3*) accounts for approximately 15% of total casein, and thus represents one of the most important proteins due to its essential role in micelle formation and stabilization [2], and thus determines the manufacturing properties of milk. Therefore, *CSN3* could be used as a positional marker gene associated with high milk production and milk composition improvement. Molecular analyses of ovine *CSN3*, mainly of exon 4, revealed synonymous and non-synonymous sequence differences [12,25,26]. However, limited information about the association of this marker with milk yield and composition in dairy sheep could be found in the literature. One positive association between *CSN3* gene polymorphism and lactose percentage has been reported in Zel sheep [20]. Staiger et al. [16] and Gras et al. [12] reported that the polymorphisms found in the *CSN3* gene did not have any significant effect on milk yield and composition traits. Hence, substantiating phenotypic data with molecular information to improve milk and its composition is critical for the future of dairy sheep industry [16]. 

The present study screens some Awassi genetic loci for possible variants in the **β-LG*, PRL*, and *CSN3* genes and establishes their frequency in commercial Awassi sheep flocks in the ultimate objective of investigating the effect of these genotypes and their interaction on milk production and composition traits. To our great knowledge, this is the first study that investigates the association of some molecular information and milk traits in the commercial Awassi flocks in Jordan.

## 2. Materials and Methods

### 2.1. Animal and Sample Collection

All experimental protocols involving animals were approved by the Animal Care and Use committee (ACUC), Jordan University and Science Technology (JUST), approval Number 16/3/3/578.

A participatory animal-breeding program was performed in the South, North, and middle of Jordan; this program was supported by Livestock and Range land research directorate of the National center for agricultural research and Extension (NCARE) (recently renamed to National Agricultural Research Center (NARC)) staff. Specific farmers were appointed before starting the program and several meetings were performed with the livestock owners. A total number of 928 ewes that belong to 9 flocks (three in each region) were targeted through the participatory animal-breeding program. 

After specifying the collaborating farms, semi-structural questionnaires were completed for each animal in the flock covering parity and age of dam, sex and type of birth of the newborns and available sires and dams in addition to all related environmental factors. All animals (n = 928) were monitored and milk data were recorded directly by three specialized teams in each region (North, middle, and South). The exhaustive milk and pedigree data however was collected from two commercial Awassi flocks, selected based on the availability and ability for collecting performance data, and located in the northern part of Jordan. Data were collected from the two farms during 5 lambing and milking seasons. The team of NARC collected the data by identifying the sires in the breeding seasons, recording lamb information (birth and weaning weights, date of birth and weaning, dam weight at lambing, milk tests as described below), after which the trained farmers continued data collection under the supervision of the livestock research director. The farmers were supplied by special separation cages. They measured milk yield with graded milk cylinders and recorded its weight with small portable digital weighing scales (up to 30 kg) or portable digital mobile weighing scales (up to 200 kg). 

Blood samples for DNA harvesting were carefully collected from the jugular vein of the 167 Awassi ewes (2 to 6 years old) that were born to 31 sires using vacuum tubes treated with 0.25% Ethylene Diamine Tetraacetic Acid (EDTA) (BD Vacutainer Systems, Plymouth, UK) and stored at −20 °C until DNA isolation.

### 2.2. Milk Samples and Analysis 

A total of 391 full lactation records on 167 ewes were collected from 2007 and 2011 from the two commercial farms identified. Milk was collected manually via hand milking by skilled workers. Through the pre-weaning period, ewes were milked once a day followed by analyzing milk amount of each ewe twice a day (morning and evening), at biweekly intervals. To measure the amount of milk produced during the suckling period (60 days), lambs were isolated from their dams 12 h before morning milking. Ewes were dried off when their milk production was reduced to less than 100 mL. The average lactation length, including suckling and milking periods was 115 days. Through the suckling period, ewes were milked once, keeping half of the milk for the lamb’s consumption, so the milk amount was multiplied by 2. While in the post-weaning period ewes were milked twice a day (morning and evening), milk amounts were averaged and multiplied by the interval between the two successive tests period then summed to obtain the total milk yield (TMY). Test-Day Milk yield (TDM) was calculated by dividing TMY by Lactation Period of each ewe. Approximately 50 mL of milk sample was collected from the morning milk of each ewe to determine basic composition of fat%, protein%, lactose%, solids-not-fat% (SNF%) and milk density g/cm^2^. Milk composition (n = 986 milk samples) was analyzed using a Milko Scan (Minor Type 78100, FOSS Electric, Hillerød, Denmark) available at JUST university. 

### 2.3. Genomic DNA Extraction and Polymerase Chain Reaction (PCR) 

Genomic DNA was extracted using Wizard Genomic DNA Extraction Kit (OMGA-Bio-Tek, Inc., Madison., WI, USA). DNA quality was tested using 1.5% agarose gel electrophoresis. Polymerase chain reaction (PCR) was used for amplifying the studied genes; using primers targeting exon II of the *β-LG*, intron 2 of PRL as shown in Table 1. Primers for CSN3 were designed, using primer 3 (http://frodo.wi.mit.edu/primer3/), to target part of the intron 3 and the totality of exon 4 and part of intron 4, using the available nucleotide sequence (Accession No.: 443394) on the NCBI GenBank database. PCR mix (HOT FIREPol DNA Polymerase; Solis BioDyne, Estonia) was carried out in a total volume of 20 μLcontaining10μL of nuclease-free water, 2 μL of genomic DNA (100 ng/ μL) as a template, 2 μL of each primer, and 4 μL (5U/µL) of Taq DNA polymerase (Eppendorf AG, Hamburg, Germany). Primer sequences, annealing temperature, and restriction enzymes used for genotyping are shown in Table 1. The PCR reaction was carried out in the following conditions of 95 °C for 5 min for initial denaturation followed by 33 cycles at 95 °C for 30 s of denaturation, 40 s annealing (Table 1) and extension each at 72 °C, and a final extension step at 72 °C for 7 min. 

### 2.4. Restriction Fragment Length Polymorphism (RFLP) Analysis

*β-LG* and *PRL* genes variants were identified by the PCR-RFLP method. The amplified *β-LG* gene fragment (301 bp) was digested by *RasI* restriction enzyme for 2 h at 37 °C. Restriction products were separated in a 2% agarose gel with ethidium bromide and visualized under ultraviolet (UV) light. The amplified *PRL* gene fragment (1209 bp) was restricted with HaeIII endonuclease at 37 °C for 3 h. The RFLP profile was visualized by the same way as for *β-LG*. 

### 2.5. Sequencing Analysis

Sequences were obtained using the same primers used for PCR amplification as shown in Table 1. The PCR products of the different genotype patterns of the *CSN3* gene were purified and sequenced by Macrogen Incorporation (Seoul, South Korea) to identify the single nucleotide polymorphisms (SNPs) found in these different genotype patterns. Ten randomly chosen PCR samples for each genotype of *β-LG* were sequenced from both directions to confirm the results obtained by PCR-RFLP technique. Sequence analysis and alignments were carried out using BioEdit program version 5.0.6. [29].

### 2.6. Statistical Analysis

The genotype and allelic frequencies of the *β-LG*, *PRL*, and *CSN3* loci were calculated using Pop-Gene 32 package version 1.31 program [30]. A chi-square (χ2) test was performed to test the goodness of fit to Hardy-Weinberg equilibrium expectations for the distribution of genotypes. The effects of genotypes of *β-LG*, *PRL*, and *CSN3*, and their interactions on the traits studied were analyzed using the least-squares method as applied in a mixed-model procedure of SAS/ STAT^®^ software (SAS Institute Inc., Cary, NC, USA, v9.1). Two statistical models were used as described below. 

The first model used for the milk production traits analysis was:

Yijklnmop = μ + BLGi+ PRLj + CSNk + Pl + Sm + SYn + βoDWo+ (BLG × PRL) ij + (BLG × CSN) ik+ (PRL × CSN) jk + eijklmnop(1)

where:

- Yijklnmop = the studied traits;
- Μ = overall mean of the total milk yield or test-day milk yield;
- BLGi = fixed effect of the ith genotype at *β-LG* locus (I = AA, AB and BB);
- PRLj = fixed effect of the jth genotype at *PRL* locus (j = AA, AB and BB);
- CSNk = fixed effect of the kth genotype at *CSN3* locus (k = TT and TC);
- Pl = fixed effect of the lth parity or number of lambing (l = 1, 2, 3, 4, 5 and 6);
- Sm= random effect of mth sires (m = 1, 2 to 31);
- SYn = fixed effect of the nth year-season of lambing (n = 2007 to 2011);
- Bo = linear regression coefficient dam weight at lambing;
- DWo = dam weight at lambing as covariate;
- (*BLG × PRL*)ij = interaction between *β-LG* genotypes and PRL genotypes (ij = AAAA, AAAB, AABB, ABAA, ABAB, ABBB, BBAA, BBAB, and BBBB);
- (*BLG* × *CSN*)ik = interaction between *β-LG* genotypes and *CSN3* genotypes (ik = AATT, AATC, ABTT, ABTC, BBTT, and BBTC);
- (*PRL* × *CSN*) jk = interaction between *PRL* genotypes and *CSN3* genotypes (jk = AATT, AATC, ABTT, ABTC, BBTT, BBTC);
- Eijklmnop = random errors with the assumption of N (0, σ2).

The second model used for the milk composition traits analysis:

Yijklmno = μ + BLGi+ PRLj + CSNk + Pl + Sm + βnTDMn + (BLG × PRL) ij + (BLG × CSN) ik + (PRL × CSN) jk + eijklmno(2)

where:

- Yijklnmo = the studied traits;
- Μ = overall mean of Fat%; protein%, SNF%, Total solids, lactose%, and density (g/cm^2^)
- BLGi = fixed effect of the ith genotype at *β-LG* locus (I = AA, AB and BB);
- PRLj = fixed effect of the jth genotype at *PRL* locus (j = AA, AB and BB);
- CSNk = fixed effect of the kth genotype at *CSN3* locus (k = TT and TC);
- Pl = fixed effect of the lth parity or number of lambing (l = 1, 2, 3, 4, 5 and 6);
- Sm = random effect of mth sires (m = 1, 2, …, 31);
- Bn = linear regression coefficient TDM.
- TDMn = TDM covariant.
- (BLG × PRL)ij = interaction between *β-LG* genotypes and PRL genotypes (ij = AAAA, AAAB, AABB, ABAA, ABAB, ABBB, BBAA, BBAB, and BBBB);
- (BLG × CSN)ik = interaction between *β-LG* genotypes and *CSN3* genotypes (ik = AATT, AATC, ABTT, ABTC, BBTT, and BBTC);
- (PRL × CSN)jk = interaction between *PRL* genotypes and *CSN3* genotypes (jk = AATT, AATC, ABTT, ABTC, BBTT, and BBTC);
- Eijklmno = random errors with the assumption of N (0, σ2).

The three-ways interaction effects among the three genes and the flock effect were removed from the model as it was not significant. For all statistical comparisons, a probability level of *p* < 0.05 was considered to be statistically significant. 

## 3. Results

### 3.1. Descriptive Statistics

The means, standard error, and coefficients of variation for milk production and composition traits are presented in Table 2. The mean milk production and composition traits showed very small standard errors but high coefficient of variations for milk production traits and fat% but relatively average CV for protein and lactose%, indicating a very diverse flock of Awassi, and further strengthening the potential for selection for both traits in these commercial flocks.

### 3.2. PCR-RLFP Assay of *β-LG* and PRL Genes

**Figure 1:**
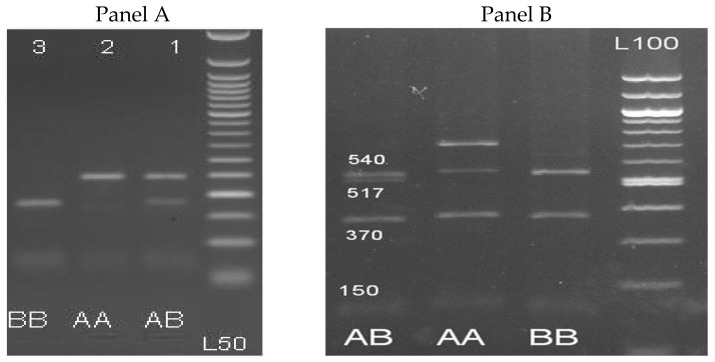
PCR-RFLP results for *β-LG* (**A**) and prolactin (**B**) genes using RsaI and HaeIII restriction enzyme respectively on 3% agarose gel. Panel A: Lanes 1, 2 and 3 are: AB (241, 175, 66 and 60 bp), AA (241 and 60 bp), and BB (175, 66 and 60 bp) genotypes, and L50: Ladder 50bp. Panel B: Lanes 1, 2 and 3 are: BB (517, 370, 147, and 151 bp), AA (540, 370, 147, and 151 bp), and AB (540, 517, 370, 147, and 151 bp) genotypes, and L100: Ladder 100 bp.

*β-LG* gene fragment was successfully amplified using PCR and resulted in a single product of 301 bp. Digestion by *RsaI* restriction enzyme revealed three genotypes designated as AA (241 and 60 bp), AB (241, 175, 66 and 60 bp) and BB (175, 66 and 60 bp) (Figure 1A) or by sequence designated as TT, TC and CC (Supplementary, Figure S2 and Table 3). For the *PRL* gene, a 1.209 bp fragment was amplified successfully. Digestion of the PCR amplified *PRL* gene by *HaeIII* restriction endonuclease is shown in Figure 1B and revealed two alleles (A and B) of three genotypes with different sizes consisting of AA (540, 370, 147, and 152 bp), AB (540, 517, 370, 147, and 152 bp) and BB (517, 370, 147, and 152 bp). 

### 3.3. Nucleotide Sequence Analysis of the *β-LG* and CSN3 Gene

A successfully amplified 680 bp specific fragment of *CSN3* gene was achieved (Supplementary, Figure S1). The sequencing of the PCR products with specific primers was performed, and the alignment and the analysis of the sequences were conducted by Bio Edit software program (Supplementary, Figure S2). In addition, showed the mutation a *Kappa casein* SNP *rs407795524* mutation located at Chr. No: 6:85316423bp and a *β-Lactoglobulin Rs430610497* mutation located at 1373 bp in Awassi sheep exon 2. 

### 3.4. Genotypic and Allelic Frequencies for *β-LG*, PRL, and CSN3 Genes

The genotypic and allelic frequencies of the *β-LG*, *PRL*, and *CSN3* genes are summarized in Table 3. The *β-LG* gene frequency had a greater prevalence of the B allele (0.58). BB and AB genotypes were predominant in the population, with frequencies of 0.32 and 0.51, respectively. The frequencies of alleles A and B at *PRL* locus were 0.82 and 0.18, respectively resulting in AA genotype being the most frequent (0.73). However, the *CSN3* gene polymorphism in this population was more restricted due to the higher frequency of the T allele (0.92), with very high frequencies for the TT (0.85) compared to TC (0.15) genotype. This result showed that T allele was of higher frequency than C allele, the assumed CC genotype of *CSN3* gene was not found in the studied Awassi population. Furthermore, the probability of deviations from the Hardy-Weinberg equilibrium for the three genes were based on chi-square test (χ^2^) and showed that all genotypic frequencies in the population were in Hardy-Weinberg equilibrium (*p* < 0.05) (Table 2). 

### 3.5. Statistical Analysis of the Effects of *β-LG*, PRL, and CSN3 Genotypes on Milk Production and Component Traits

The fixed effects on milk production and some milk composition traits are presented in Table 4 and showed that sire, parity, and year-season of lambing had highly significant effects on milk production traits (*p* < 0.05). However, the *β-LG* and *CSN3* gene had no effect (*p* > 0.05) on milk production traits (TMY and TDM), in contrast to the *PRL* gene (*p* < 0.05). Milk production traits were significantly affected by the interaction between *β-LG* and *PRL* genes (*p* < 0.05), while milk production did not differ between the pairs of other combination genes genotypes interactions (**β-LG* × CSN3*, and PRL × *CSN3* genes) (*p* > 0.05). 

Furthermore, the *β-LG* gene had significant effects on all milk composition traits (*p* < 0.05). *PRL* gene significantly (*p* < 0.05) affected all milk composition traits except fat%. Out of all the studied composition traits, *CSN3* gene had an effect only on SNF% (*p* < 0.05) (Table 4). Also, sire had highly significant effects on all milk composition traits (*p* < 0.05), while the effect of parity was not significant (*p* > 0.05). Combined gene genotypes also showed significant (*p* < 0.05) interaction effects between *β-LG* and *PRL* on all milk composition traits, while the interactions between *β-LG* and *CSN3* did not show significant effects (*p* > 0.05). This interaction can be clearly seen in Table 4, where the interaction between *PRL* and *CSN3* gene significantly altered (*p* < 0.05) only fat% and SNF%.

### 3.6. Effects of *β-LG*, PRL, and CSN3 Genotypes on Milk Production Traits and Their Interactions

The effect of *β-LG*, *PRL*, and CSN3 genotypes on milk production of Awassi sheep are shown in Table 5. Although the results indicated that the AA and BB genotypes produced the highest milk production (TMY and TDM) compared to the AB genotype, no significant differences (*p* > 0.05) were observed between the genotype groups of *β-LG*. For *PRL* gene, AA genotype was associated with the highest milk production compared to AB genotype, while no significant differences (*p* > 0.05) in milk production were observed between the AA and BB genotypes. The *CSN3* gene showed no significant association with milk production traits (Table 5).

The least square means (±SE) for the effect of interaction between *β-LG* × *PRL* genotypes on milk production traits are shown in Table 5. The results for the **β-LG* × PRL* indicated that AA × BB was significantly associated with maximum TMY and TDM compared to the other genotypes.

### 3.7. Effects of *β-LG*, PRL, and CSN3 Genotypes on Milk Composition Traits and Their Interactions

Table 6 illustrates the results obtained for the effect of **β-LG*, PRL* and *CSN3* genotypes on milk composition traits of Awassi sheep and the different interactions between the various genotypes. The AA and BB genotypes of *β-LG* gene were associated with the highest fat levels (6.63 and 6.30, respectively), while AB genotype with the lowest (5.31) (*p* < 0.05). The BB genotype of *β-LG* gene also showed significant (*p* < 0.05) association with the highest SNF%, protein% and lactose% compared to the AA and AB genotypes. 

Specifically, the *β-LG* BB genotype was associated with the highest milk density compared to AA genotype (35.4 ± 0.71, 33.0 ± 0.61 g/cm^2^, *p* < 0.05, respectively), but not to genotype AB (34.1 ± 0.87 g/cm^2^, *p* > 0.05). In relation to the *PRL* gene, no significant differences among *PRL* genotypes were found in fat%, while the AA and BB genotype were associated with the high SNF% compared with AB genotype. Greater protein percentages were recorded for AA compared to AB genotypes, but not BB. The highest lactose percentage was found in the BB genotype of *PRL* gene (5.35 ± 0.18%) compared to the AA and AB genotypes (5.02 ± 0.08%, 4.90 ± 0.09%, respectively). Moreover, AA and BB genotypes produced the highest milk density compared to AB genotype. For the *CSN3* gene, TT genotype was associated with the highest levels of SNF% (10.1 ± 0.19%), while the TC was associated with the lowest (9.45 ± 0.15%). Nonetheless, no significant differences were found among *CSN3* genotypes in milk components including fat%, protein%, lactose%, and milk density (*p* > 0.05). 

Compared to their other respective genotypes; AA × BB genotype (*β-LG* × *PRL*) showed the highest fat%, BB × BB genotype (*β-LG* × *PRL*) recorded the highest SNF%, protein%, lactose% and milk density, and BBxTT genotype (PRL × *CSN3*) scored the highest fat% and SNF%. Interaction differences among *PRL* × *CSN3* genotypes in protein% and lactose% and milk density (*p* > 0.05) were not significant. 

## 4. Discussion

This study reports the association between *β-LG*, *PRL*, and *CSN3* genes and milk production and composition of Awassi sheep. These genes were chosen because of their direct involvement in the growth and development of the mammary gland, maintenance of milk secretion, and synthesis of milk [2,12,14,15,22,31]. The genes are also located in the region of QTL influencing milk quantity and quality [12,14,15,22,23]. This study revealed that in Awassi sheep, these genes present different allelic frequencies and genotypes (Table 2). 

*β-LG* locus showed a higher frequency of allele B (0.58) than allele A (0.42) in Awassi sheep. Similar results were found in Chios sheep breed [19], Racka Sheep [32], Rusty Tsigai breed [33], Zel breed [20], Polish Merino breed [34], and Awassi breed [3,35]. No evidence was found for C allele in our study, which is considered a rare variant detected only in a few breeds such as Turcana, Racka, Tsigai, Karakul of Botosani, Transylvanian Merino, Merinoland, and Hungarian Merino [15,36]. 

In agreement with other studies, the frequency of *PRL* A allele (0.82) was more than B allele (0.18); Allele frequency was found at 0.64 in Spanish Merino sheep [14], 0.53 in Black Head sheep breed [12], 0.75 in Awassi sheep [37], 0.64 in Serra da Estrela, 0.57 in White Merino, and 0.72 in Black Merino [38]. 

However, the gene frequency for the CSN3 locus in Awassi sheep reported in this study differed from East Friesian sheep [16] and Black Head sheep [12] where the T and C alleles were of equal frequencies. 

The findings presented in this paper indicated that *β-LG* gene polymorphisms are not associated with milk production traits in Awassi sheep (Table 4). These results are in agreement with previous studies reported by Padilla et al. [14] in Spanish Merino sheep, Triantaphyllopoulos et al. [19] in Karagouniko and Chios sheep breeds, Giambra et al. [39] in East Friesian Dairy and Lacaune sheep, Kaweka and Radko [34] in Polish Mountain, Polish Merino, East Friesian and Bergschaf, and Staiger et al. [16] in East Friesian sheep where the effect of *β-LG* variants on milk yield was not indicated. Although *β-LG* gene did not show any associations with high milk yield, inverse response over milk composition must be considered in marker assisted selection (MAS) strategy. We found that the AA and BB genotypes of *β-LG* gene were associated with the highest fat percentage compared to AB genotype. Some previous studies reported significant effects of *β-LG* on fat content in milk of different sheep breeds. Positive effects on milk fat content varied depending on sheep breed and *β-LG* genotype; *β-LG* BB genotype had a significant effect on milk fat percentage in Awassi sheep [40], AA and AB genotypes in Italian Altamurana and Leccese sheep [41], AA genotype in Merino sheep [42] and East Friesian Dairy sheep [39], AB genotype in Zel breed [20]. A recent study conducted by Padilla et al. [14] on the Spanish Merino sheep showed that *β-LG* A allele had positive significant effects on fat percentage.

We also found that the BB variant of the *β-LG* gene had a very large positive effect on protein%, SNF%, lactose%, and milk density compared to AB and AA genotypes (Table 4). These results were consistent with many previous studies where a strong association of the BB variant with protein percentage [43,44] and lactose content [19,20,45], but inconsistent in regard to SNF% [34,41,44,46] and milk density [20]. 

When analyzing the PRL genotypes effects on milk production traits, the AA genotype was associated with the highest milk production (Table 5). This result is consistent with some published results [12,13,16] who reported a significant difference confirming the superiority of the *PRL* AA genotype in milk yield. However, fat percentage was not significantly affected by the *PRL* genotype (Table 6) consistent with some published reports [14,38]. Moreover, contrary to available findings of BB genotype’s superiority for protein percentage [12,14,38], our study found a stronger correlation with the AA genotype of the *PRL* gene compared to the AB and BB genotypes in Awassi ewes. Another study using Sakiz ewes, Ozmen and Kul [13] found that heterozygous AB ewes produced the highest milk protein percentage when compared to homozygous AA and BB genotypes animals, while there were no significant differences in protein percentage according to different genotypes in Akkaraman and Awassi ewes. *PRL* AA and BB genoty [13] pe also showed positive effects on SNF% and milk density (Table 6); however, according to the literature, no other studies examined this association. This study also showed that the BB genotype of the *PRL* gene particularly was associated with higher production of lactose in milk compared with the AA and AB genotypes. In contrast to these results, Ozmen and Kul [13] reported lack of association between lactose content and PRL genotypes in Sakiz, Akkaraman, and Awassi ewes. 

Our results did not show any significant effect of the *CSN3* variants on milk production traits as seen in the milk yield of Black Head sheep [12] and East Friesian sheep [16]. We found no significant effect of *CSN3* variants on fat, protein, lactose content, and milk density, whereas SNF content was positively affected by only the TT genotype (Table 6). Gras et al. [12] also reported no associations when his team examined the influence of *CSN3* polymorphism on fat and protein content in Black Head sheep. On the other hand, Yousefi et al. [20] reported that the k1 pattern of the *CSN3* locus affected only lactose percentage in milk and milk density in Zel sheep. 

The interesting portion of this project was the combined genotype effect on milk production and composition; genotype combination reflects the interactions of multiple genes effects in a certain quantitative trait [47]. Our study only found a significant impact of the interaction of β-LG*PRL genotypes on milk production (Table 6), while PRL *× CSN3* and *β-LG* × *CSN3* genotypes showed minimal combined effects. (*p* < 0.05). Mile production was highest in Awassi ewes of AA × BB (*β-LG* × PRL) combined genotypes compared to the ABxBB genotype. While milk composition only improved in the *β-LG* × PRL and PRL × CSN3 genotypes compared to *β-LG* × CSN3 (*p* < 0.05). The statistical results show that ewes of AA × BB genotype combination had the highest fat%, and BB × BB genotype combination had the highest SNF%, protein%, lactose% and milk density compared to other genotypes (Table 6). The highest fat% and SNF% were recorded for the BB × TT genotype (PRL × *CSN3*) compared to the other genotypes. 

## 5. Conclusion

The findings presented in this paper indicated that *β-LG* gene polymorphisms are not associated with milk production traits, but rather with varying fat%, protein%, SNF%, lactose%, and even density. PRL gene polymorphism was associated with positively with SNF%, lactose% and milk density but not fat% or protein%. Furthermore, there was no *CSN3* variants effects on milk production or composition traits in Awassi ewes. 

The interesting portion of this project was the combined genotype effect on milk production and composition where we showed a significant impact of the interaction of *β-LG* × *PRL* genotypes on milk production and of PRL × *CSN3* on fat% and SNF%, while minimal combined effects for PRL × *CSN3* and **β-LG* × CSN3* genotypes were detected. 

Although our study showed the great potential of these three genes and their variants on improving milk yield and composition traits, the interaction and combined effects of these genes should be studied further in order to incorporate them in breeding strategies to improve milk production and composition of Awassi sheep.

## Figures and Tables

**Table 1:** Primer information and restriction information for genes of interest.

Gene	Primers (5′→3′)		TM (°C)	PCR Product (bp)	RE	Reference
Beta-lactoglobulin (*β-LG*)	F	CTCTTTGGGTTCAGTGTGAGTCTTG	58	301	RsaI	[27]
	R	CACCATTTCTGCAGCAGGATCTC				
Prolactin (*PRL*)	F	ACCTCTCCTCGGAAATGTTCA	56	1209	HaeIII	[28]
	R	GGGACACTGAAGGACCAGAA				
Kappa Casein (*CSN3*)	F	CTGGGTTCACTATTCCCAATG	57	680	*	Accession # 443394
	R	TTGCTCATTTACCTGCGTTG				

TM: Annealing temperature, RE: restriction enzyme used for Restriction Fragment Length Polymorphism (RFLP) and * sequence only not RFLP

**Table 2:** Descriptive statistics of milk production and milk composition traits.

Milk Trait	No. Records	Mean	SE	CV (%)
Milk production (Kg)				
TMY	391	96.8	2.60	53.0
TDM	391	0.882	0.02	45.0
Milk composition				
Fat%	917	5.80	0.05	25.7
SNF%	986	9.74	0.03	8.30
Protein%	986	3.90	0.02	13.0
Lactose%	986	5.10	0.02	13.9
Density g/cm^2^	986	34.3	0.10	9.4

TMY: Total Milk Yield; TDM: Test-Day Milk; SNF: Soluble-Not-Fat; SE: Standard Error; CV: Coefficient of Variation (%).

**Table 3:** Allelic and genotypic frequencies for **β-LG*, PRL*, and *CSN3* genes in Awassi sheep.

Gene ^1^	Genotype	Observed Number	Expected Number	Genotype Frequency	Allele	Allele Frequency	Value of x^2^ Test
*β-LG* (n = 159)	AA	27	28.7	0.17	A	0.42	0.29 ^ns^
	AB	81	77.7	0.51	B	0.58	
	BB	51	52.7	0.32			
*PRL* (n = 158)	AA	115	107	0.73	A	0.82	19.2 ^ns^
	AB	30	46.1	0.19	B	0.18	
	BB	13	5	0.08			
*CSN3* (n =156)	TT	132	132.9	0.85	T	0.92	1.1 ^ns^
	TC	24	22.1	0.15	C	0.08	

^1^*β-LG* = β-lactoglobulin; *PRL* = prolactin; *CSN3* = κ-casein; n: number of animals; ns: Non-significant (*p* > 0.05), n = number of genotyped individuals

**Table 4:** *p*-values for milk production and composition traits.

	Trait	Milk Production ^1^		Milk Components ^2^				
Factors		TMY (kg)	TDM (kg)	Fat%	SNF%	Protein%	Lactose%	Density, g/cm^2^
*β-LG*		0.175	0.134	0.047	<0.0001	0.046	0.03	0.002
*PRL*		0.034	0.011	0.761	0.001	0.035	0.05	0.005
CSN3		0.812	0.275	0.172	0.048	0.424	0.104	0.541
Sire		<0.0001	<0.0001	<0.0001	<0.0001	0.019	0.035	0.0004
Parity		0.004	0.005	0.056	0.412	0.389	0.266	0.665
Year		0.003	0.002					
*β-LG* × *PRL*		0.039	0.031	0.001	<0.0001	0.008	0.035	0.05
*β-LG* × *CSN3*		0.874	0.221	0.417	0.899	0.784	0.949	0.496
*PRL* × *CSN3*		0.177	0.104	0.002	0.02	0.228	0.115	0.767
Dam weight at lambing		0.299	0.009					
Test-day milk				0.004	0.002	0.0004	0.481	<0.0001

^1^ mixed-model analysis of fixed effects; ^2^ analysis of some milk component factors. TMY: total milk yield; TDM: test-day milk; SNF: Solids-Non-Fat; *β-LG* = β-lactoglobulin; *PRL* = prolactin; *CSN3* = κ-casein

**Table 5:** Effect of Beta lactoglobuline (**β-LG*)*, Prlactin (*PRL)*, and *Kappa casein (CSN3)* genotypes and significant interaction effects on milk production traits in Awassi sheep.

Gene	Genotype	N	Trait Least Square Means (±SE)	
			TMY (Kg)	TDM (Kg)
*β-LG*	AA	51	100.4 ± 13.4	0.718 ± 0.10
	AB	145	72.2 ±15.7	0.606 ± 0.10
	BB	96	93.2 ± 12.6	0.801 ± 0.10
*PRL*	AA	197	102.4 ± 9.86 ^a^	0.814 ± 0.08 ^a^
	AB	68	71.4 ± 12.6 ^b^	0.540 ± 0.10 ^b^
	BB	27	92.0 ± 14.7 ^ab^	0.770 ± 0.11 ^a^
*CSN3*	TT	240	90.1 ± 11.8	0.762 ± 0.09
	TC	52	87.1 ± 11.5	0.654 ± 0.09
**β-LG* × PRL*	AAAA	29	96.5 ± 13.0 ^b^	0.780 ± 0.10 ^ab^
	AAAB	12	65.0 ± 23.0 ^bc^	0.359 ± 0.17 ^c^
	AABB	10	139.7 ± 25.2 ^a^	1.02 ± 0.19 ^a^
	ABAA	106	99.2 ± 17.3 ^ab^	0.834 ± 0.13 ^ab^
	ABAB	31	72.6 ± 19.3 ^bc^	0.578 ± 0.15 ^c^
	ABBB	8	44.9 ± 18.4 ^c^	0.406 ± 0.14 ^c^
	BBAA	62	111.6 ± 11.1 ^ab^	0.829 ± 0.08 ^ab^
	BBAB	25	76.8 ± 15.4 ^bc^	0.684 ± 0.12 ^b^
	BBBB	9	91.3 ± 27.6 ^ab^	0.891 ± 0.21 ^ab^

Within the same column different letters indicate significant differences *p* < 0.05.

**Table 6:** Effect of *β-LG*, *PRL*, and *CSN3* genotypes and their interacting effects on milk composition traits in Awassi sheep.

Gene	Genotype	N	Traits Least Square Means (±SE)				
			Fat%	SNF%	Protein%	Lactose%	Density, g/cm^2^
*β-LG*	AA	112	6.63 ± 0.29 ^a^	9.50 ± 0.15 ^b^	3.90 ± 0.10 ^b^	4.99 ± 0.14 ^b^	33.0 ± 0.61 ^b^
	AB	376	5.31 ± 0.43 ^b^	9.39 ± 0.22 ^b^	3.66 ± 0.14 ^b^	4.88 ± 0.20 ^b^	34.1 ± 0.87 ^ab^
	BB	277	6.30 ± 0.35 ^a^	10.4 ± 0.18 ^a^	4.13 ± 0.12 ^a^	5.39 ± 0.16 ^a^	35.4 ± 0.71 ^a^
*PRL*	AA	554	6.13 ± 0.17	9.86 ± 0.08 ^a^	4.00 ± 0.06 ^a^	5.02 ± 0.08 ^b^	34.2 ± 0.34 ^a^
	AB	159	5.96 ± 0.19	9.44 ± 0.10 ^b^	3.79 ± 0.06 ^b^	4.90 ± 0.09 ^b^	32.9 ± 0.40 ^b^
	BB	52	6.15 ± 0.38	9.96 ± 0.19 ^a^	3.91 ± 0.13 ^ab^	5.35 ± 0.18 ^a^	35.3 ± 0.77 ^a^
*CSN3*	TT	624	6.49 ± 1.45	10.1 ± 0.19 ^a^	3.98 ± 0.12	5.32 ± 0.17	34.5 ± 0.75
	TC	141	5.67 ± 1.05	9.45 ± 0.15 ^b^	3.82 ± 0.10	4.86 ± 0.14	33.7 ± 0.62
*β-LG* × PRL	AAAA	56	6.38 ± 0.34 ^bc^	9.77 ± 0.17 ^b^	4.05 ± 0.11 ^b^	4.87 ± 0.16 ^c^	33.6 ± 0.70 ^b^
	AAAB	29	5.56 ± 0.45 ^c^	8.63 ± 0.21 ^c^	3.47 ± 0.14 ^c^	4.46 ± 0.19 ^d^	30.2 ± 0.84 ^c^
	AABB	27	7.95 ± 0.82 ^a^	10.1 ± 0.42 ^b^	4.18 ± 0.28 ^ab^	5.66 ± 0.39 ^ab^	35.2 ± 1.71 ^ab^
	ABAA	284	5.97 ± 0.24 ^c^	9.87 ± 0.11 ^b^	3.99 ± 0.08 ^b^	5.06 ± 0.10 ^bc^	34.6 ± 0.46 ^b^
	ABAB	78	6.67 ± 0.30 ^b^	9.97 ± 0.15 ^b^	3.97 ± 0.10 ^b^	5.24 ± 0.14 ^b^	34.5 ± 0.61 ^b^
	ABBB	14	3.28 ± 1.11 ^d^	8.33 ± 0.57 ^c^	3.02 ± 0.38 ^c^	4.35 ± 0.50 ^cd^	33.3 ± 2.30 ^bc^
	BBAA	214	6.03 ± 0.18 ^c^	9.92 ± 0.09 ^b^	3.96 ± 0.06 ^b^	5.12 ± 0.08 ^bc^	34.6 ± 0.36 ^b^
	BBAB	52	5.63 ± 0.38 ^c^	9.72 ± 0.15 ^b^	3.91 ± 0.10 ^b^	4.99 ± 0.14 ^bc^	34.1 ± 0.59 ^b^
	BBBB	11	7.23 ± 1.03 ^ab^	11.5 ± 0.52 ^a^	4.53 ± 0.35 ^a^	6.05 ± 0.48 ^a^	37.4 ± 2.10 ^a^
PRL × CSN3	AATT	478	6.13 ± 0.18 ^b^	9.89 ± 0.08 ^b^	3.95 ± 0.05	5.11 ± 0.07	34.5 ± 0.32
	AATC	75	6.12 ± 0.27 ^b^	9.82 ± 0.13 ^bc^	4.05 ± 0.09	4.93 ± 0.12	33.9 ± 0.54
	ABTT	122	5.32 ± 0.22 ^c^	9.29 ± 0.11 ^d^	3.68 ± 0.46	4.83 ± 0.17	32.9 ± 1.02
	ABTC	37	6.59 ± 0.32 ^b^	9.59 ± 0.16 ^c^	3.89 ± 0.11	4.68 ± 0.15	32.9 ± 1.45
	BBTT	23	8.02 ± 1.11 ^a^	11.0 ± 0.57 ^a^	4.31 ± 0.38	6.03 ± 1.12	36.2 ± 2.30
	BBTC	29	4.29 ± 0.75 ^d^	8.93 ± 0.38 ^d^	3.51 ± 0.62	4.68 ± 0.35	34.5 ± 1.55

Within the same column different letters indicate significant differences *p* < 0.05.

## Supplementary Materials

The following are available online at https://www.mdpi.com/2076-2615/9/6/382/s1, Figure S1: κ-casein (CSN3) gene PCR product, lanes 1 and 2 is the 680bp PCR product and L50: Ladder 50bp, Figure S2: Sequencing of *Kappa **Case**in* and β-Lactoglobulin in Awassi sheep. Panel I. *Kappa casein* SNP rs407795524 mutation detected by sequencing in Awassi sheep A: TC, B: TT and C: CC located at Chr. No: 6:85316423bp; Panel II: Rs430610497 mutation that located at 1373bp in Awassi sheep exon 2 of *β-Lactoglobulin* gene A: Heterozygous TC, B: CC and C: TT genotype.
