# Supplementary material for: Effect and Interaction of β-Lactoglobulin, Kappa Casein, and Prolactin Genes on Milk Production and Composition of Awassi Sheep
Source: Animals (Basel). 2019 Jun 21;9(6):382. doi: 10.3390/ani9060382 (PMC6617529; doi:10.3390/ani9060382)

Supplemental material

Figure S1: κ-casein (CSN3) gene PCR product, lanes 1 and 2 is the 680bp PCR product and L50: Ladder 50bp

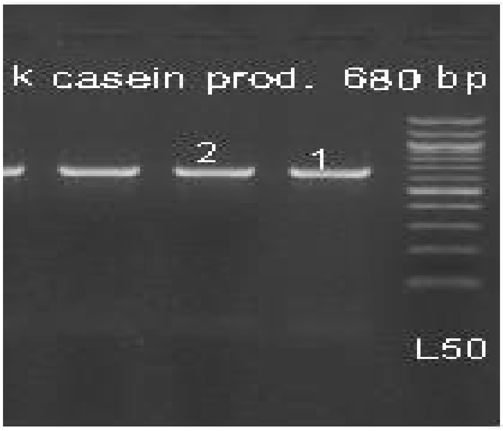

Figure S2: Sequencing of Kappa Casein and β-Lactoglobulin in Awassi sheep. Panel I. Kappa casein SNP rs407795524 mutation detected by sequencing in Awassi sheep A: TC, B: TT and C: CC located at Chr. No: 6:85316423bp; Panel II: Rs430610497 mutation that located at 1373bp in Awassi sheep exon 2 of β-Lactoglobulin gene A: Heterozygous TC, B: CC and C: TT genotype.

I. Kappa Casein rs407795524 II. β-Lactoglobulin Rs430610497

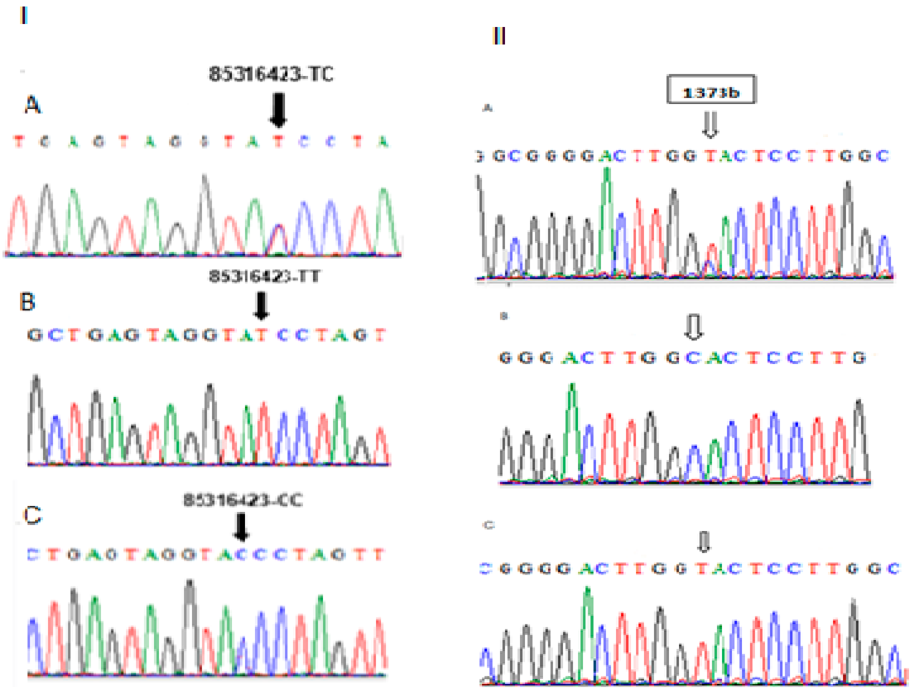

Supplement: Supplementary file 1 [file animals-09-00382-s001.pdf]
